# Sex differences in escalation to atrioventricular node ablation following catheter ablation for atrial fibrillation

**DOI:** 10.1007/s10840-026-02395-7

**Published:** 2026-07-01

**Authors:** Rosie Oatham, Gregory Y. H. Lip, Mark T. Mills

**Affiliations:** 1 Department of Cardiology, Northern General Hospital, Sheffield Teaching Hospitals NHS Foundation Trust, Sheffield, S5 7AU UK; 2 Department of Cardiology, Liverpool Heart & Chest Hospital, Thomas Drive, Liverpool, L14 3PE UK; 3 Liverpool Centre for Cardiovascular Science at University of Liverpool, Liverpool John Moores University and Liverpool Heart & Chest Hospital, Liverpool, UK; 4 Department of Clinical Medicine, Aalborg University, Aalborg, Denmark; 5 Department of Cardiology, Lipidology and Internal Medicine with Intensive Coronary Care Unit, Medical University of Bialystok, Bialystok, Poland

**Keywords:** Atrial fibrillation, Catheter ablation, Atrioventricular node, Pulmonary vein isolation, Sex differences

## Abstract

**Background:**

Sex-based differences in atrial fibrillation (AF) symptom burden and response to rhythm control strategies are recognised. Whether female and male patients differ in escalation to atrioventricular (AV) node ablation after AF catheter ablation remains uncertain.

**Objective:**

To evaluate sex differences in AV node ablation following AF ablation in a large real-world cohort.

**Methods:**

Adults undergoing first-time AF ablation between 2015 and 2024 were identified from a global federated research network. Patients with prior AV node ablation or cardiac implantable electronic devices were excluded. Female and male patients were compared before and after 1:1 propensity score matching for demographics, AF type, heart failure phenotype, co-morbidities, medication use and prior cardioversion. The primary outcome was AV node ablation within 5 years. Secondary outcomes included repeat AF ablation, electrical cardioversion and all-cause mortality. Pre-specified falsification endpoints (pneumonia and intestinal obstruction) were assessed to evaluate residual confounding.

**Results:**

Before matching (28,004 female; 52,137 male), AV node ablation occurred in 2.5% of female and 1.1% of male patients (risk ratio [RR] 2.31, 95% CI 2.07–2.58). After matching (24,377 female and 24,377 male), baseline characteristics were well balanced, yet AV node ablation remained more frequent in female than male patients (2.3% vs. 1.2%; RR 1.94, 95% CI 1.68–2.23). Female patients were also more likely to undergo repeat AF ablation (14.7% vs. 14.1%; RR 1.04, 95% CI 1.01–1.08), whilst mortality was modestly lower (4.5% vs. 4.9%; RR 0.91, 95% CI 0.84–0.99; *p* = 0.027). Falsification endpoints did not differ between groups after matching.

**Conclusion:**

Female patients were more likely than male patients to undergo AV node ablation following AF ablation despite robust adjustment, suggesting important sex-based differences in downstream AF management.

**Graphical abstract:**

**Figure:**
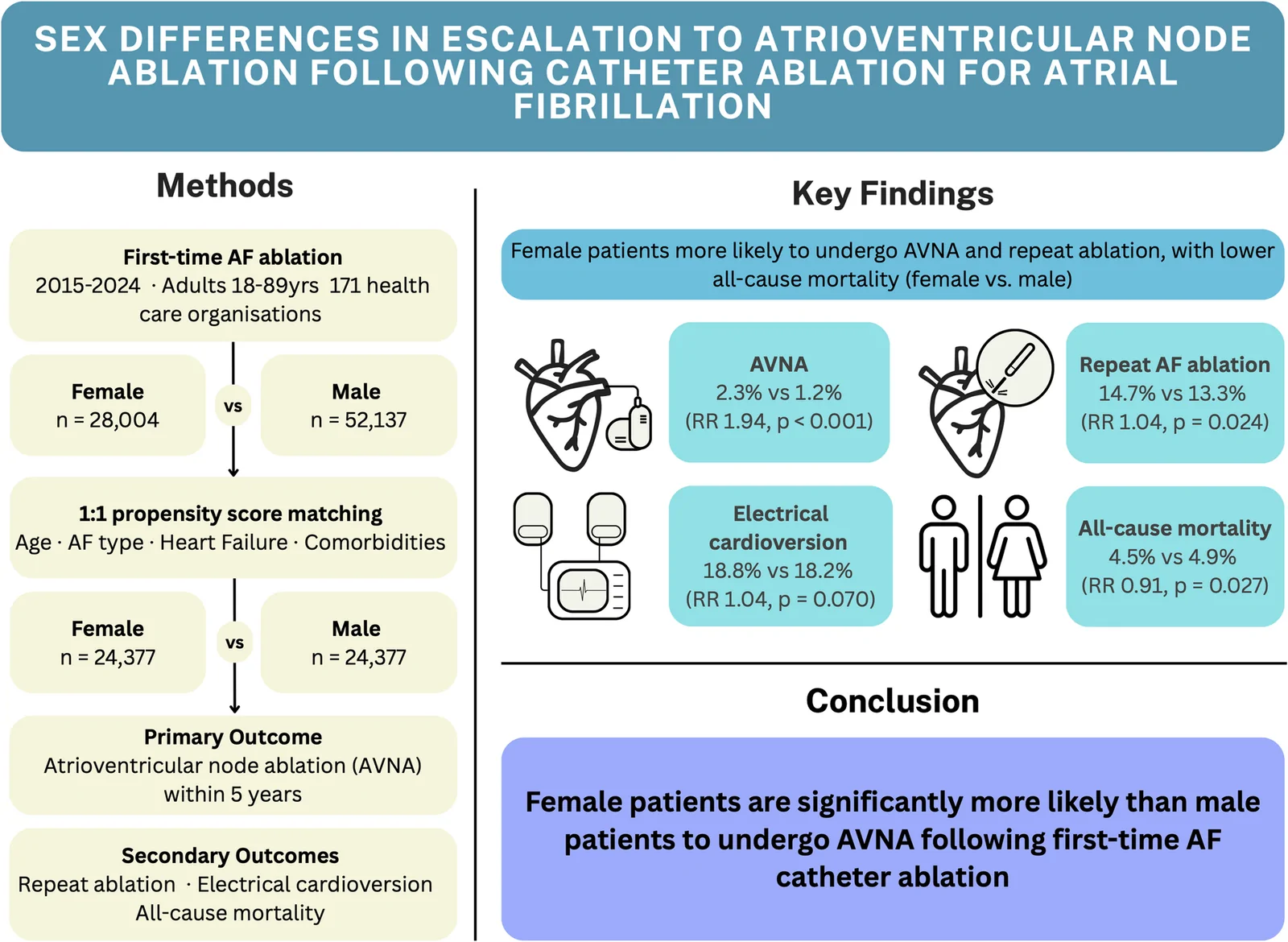
Central illustration

**Supplementary Information:**

The online version contains supplementary material available at https://doi.org/10.1007/s10840-026-02395-7.

## Introduction

Sex-differences in the epidemiology, management and outcomes of atrial fibrillation (AF) are well-established [1–5]. Women with AF are typically older at presentation with a higher burden of symptoms, lower quality of life (QoL) and increased risk of stroke and cardiovascular mortality [2–4, 6–13]. Despite this, referral for specialist management is often delayed [14–16]. Evidence suggests that women have a greater degree of adverse events with anti-arrhythmic drug (AAD) therapy [11, 17, 18], and yet they are less likely to be offered invasive therapies, including electrical cardioversion and catheter ablation [2, 16, 19]. Additionally, they may experience more procedural complications, with often less durable results [15, 20–25]. Understanding the basis for such disparities is essential to deliver equitable care.

Contemporary AF management focuses on a patient-centred model, with guidelines highlighting the importance of optimal treatment to reduce symptoms, including catheter ablation [26]. Where rate control is insufficient or AF is refractory to treatment, atrioventricular node ablation (AVNA) with permanent pacemaker implantation (“pace-and-ablate” strategy) is recognised as a safe and effective treatment, with low long-term mortality and improvement in both symptoms and QoL [27–33]. AVNA provides rate control and regularisation of the R-R interval, which is of particular benefit in the sub-set of patients with symptomatic permanent AF and heart failure (HF). In the APAF-CRT randomised controlled trial, AVNA combined with cardiac resynchronisation therapy (CRT), and optimisation of biventricular pacing, was superior to pharmacological therapy for reducing all-cause mortality and hospitalisation for HF [28]. It is however recognised that rate control with right ventricular (RV) pacing alone can result in pacing-related cardiomyopathy, which may offset the benefits of AVNA for the management of AF [34].

Despite the established evidence base, the question of whether sex influences the escalation to AVNA after first-time catheter ablation for AF remains unanswered. It is plausible that the documented disparities between men and women with AF may influence decisions to proceed with a pace-and-ablate strategy. Existing trials were not designed to address sex-specific differences in the indication for, or utilisation of AVNA. This study therefore aims to determine whether sex-based differences exist in escalation to AVNA following first-time AF ablation, and to identify potential inequities in treatment pathways that may inform more tailored and equitable management strategies.

## Methods

### Study design, setting and participants

This study was a retrospective, multi-centre, observational cohort study utilising data from the TriNetX Research Network, a large international federated analytics platform that provides access to anonymised electronic health record data from more than 250 million individuals across over 200 healthcare organisations in 19 countries [35, 36]. The network includes data from both hospital-based and community healthcare settings. Participating institutions contribute datasets that are mapped to standardised clinical coding systems, including the *International Classification of Diseases*,* Tenth Revision* (ICD-10), *Current Procedural Terminology* (CPT), *Systematized Nomenclature of Medicine Clinical Terms* (SNOMED) and *RxNorm * [37, 38]. Data undergo internal validation and quality assurance processes coordinated by TriNetX in collaboration with contributing sites [39]. Only de-identified, aggregated data are available for analysis, and institutional identities are not disclosed to safeguard privacy. In accordance with the federated structure of the platform, separate ethical approval was not required. The study was conducted in line with STROBE reporting recommendations.

We identified adults aged 18–89 years undergoing first-time catheter ablation for AF between 1st January 2015 and 31st December 2024. AF ablation was identified using the CPT code 93656. To identify first-time AF ablation procedures, patients with any prior recorded AF ablation (CPT 93656) before the index ablation date were excluded from cohort inclusion. Patients with a history of prior AVNA, permanent pacemaker implantation, or CRT were excluded. Baseline demographic characteristics, co-morbidities, and medication use at the time of AF ablation were extracted. All diagnostic and procedural codes used are detailed in the Supplementary Materials (Appendix 1). The database query was last performed on 11th February 2025.

### Study outcomes

The primary outcome was AVNA occurring within 5 years following the index AF ablation. AVNA was identified using relevant procedural codes within the TriNetX platform. Time-to-event was calculated from the date of the index AF ablation to the first recorded AVNA.

Secondary outcomes included repeat AF ablation, electrical cardioversion and all-cause mortality within 5 years of index AF ablation. Repeat AF ablation was defined using CPT codes 93656 and/or 93657 occurring after the index procedure. Electrical cardioversion was identified using corresponding procedural codes. Mortality data were captured within the electronic health record network and analysed as all-cause mortality.

To assess for residual confounding and evaluate the adequacy of propensity score matching, two pre-specified falsification endpoints were examined: pneumonia and intestinal obstruction. These outcomes were selected a priori as clinical events not plausibly related to patient sex in the context of AF ablation strategy.

Patients were followed from the date of index AF ablation until the occurrence of the outcome of interest, death, loss to follow-up within the network, or completion of 5 years of follow-up, whichever occurred first.

The healthcare codes used to identify these outcomes are summarised in the Supplementary Materials (Appendix 1).

### Statistics analysis

All analyses were conducted within the TriNetX analytics platform. Continuous variables are reported as mean ± standard deviation and categorical variables as counts and percentages. Between-group comparisons were performed using independent-sample t tests for continuous variables and χ² tests for categorical variables, as appropriate.

To minimise baseline imbalance between female and male patients, 1:1 propensity score matching (PSM) was undertaken. Propensity scores were generated using logistic regression within the TriNetX platform, incorporating demographic variables, AF subtype, HF phenotype, cardiovascular and non-cardiovascular co-morbidities, medication use and prior cardioversion. Greedy nearest-neighbour matching was applied with a caliper width of 0.1 pooled standard deviations. Covariate balance was assessed using standardised mean differences (SMD), with values < 0.1 considered indicative of adequate balance.

The primary and secondary outcomes were analysed over a follow-up period of up to 5 years. Cumulative incidence was estimated using Kaplan–Meier survival analysis and differences between groups were assessed using the log-rank test. Effect estimates are reported as risk ratios (RR) with 95% confidence intervals (CI). A two-sided P value < 0.05 was considered statistically significant. Unless otherwise specified, results are presented as female versus male comparisons.

## Results

### Demographics and matching

A total of 80,141 patients undergoing first-time catheter ablation for AF between 2015 and 2024 without prior AVNA, permanent pacemaker implantation, or CRT were identified from the TriNetX Global Collaborative Network (female: 28,004; male: 52,137). Baseline patient characteristics are summarised in Table 1, both before and after PSM. After 1:1 PSM, the final matched cohorts comprised 24,377 patients in each group (48,754 patients in total).

**Table 1:** Patient characteristics before and after propensity score matching

	Before PSM				After PSM			
	Female (*n* = 28,004)	Male (*n* = 52,137)	*p*-value	SMD	Female (*n* = 24,377)	Male (*n* = 24,377)	*p*-value	SMD
Demographics								
Age (years), mean ± SD Race (%)	67.2 ± 10.1	63.8 ± 10.7	**< 0.001**	0.33	66.5 ± 10.2	66.5 ± 9.8	0.672	< 0.01
White	82.2	83.6	**< 0.001**	0.03	82.4	82.6	0.642	< 0.01
Black	7.0	5.2	**< 0.001**	0.07	6.4	6.3	0.424	0.01
Asian	2.9	2.7	0.126	0.01	3.0	2.8	0.402	0.01
Co-morbidities (%)								
Persistent atrial fibrillation	41.2	47.0	**< 0.001**	0.12	41.3	40.8	0.250	0.01
Systolic heart failure	12.0	16.6	**< 0.001**	0.13	12.3	12.2	0.600	0.01
Diastolic heart failure	16.0	10.2	**< 0.001**	0.15	13.4	13.1	0.330	0.01
Type 2 diabetes mellitus	20.9	21.1	0.390	0.01	20.3	19.9	0.205	0.01
Cerebral infarction	6.1	5.0	**< 0.001**	0.05	5.6	5.4	0.322	0.01
Acute myocardial infarction	7.2	8.3	**< 0.001**	0.04	7.1	6.9	0.558	0.01
Chronic ischaemic heart disease	29.3	37.0	**< 0.001**	0.17	29.8	29.5	0.513	0.01
Hypertension	64.3	62.9	**< 0.001**	0.03	62.3	61.4	**0.042**	0.02
Mitral valve regurgitation	23.3	19.3	**< 0.001**	0.10	21.2	21.0	0.491	0.01
Chronic kidney disease	11.7	12.2	**0.025**	0.02	11.3	11.2	0.657	< 0.01
Emphysema	3.6	3.5	0.307	0.01	3.5	3.4	0.901	< 0.01
Pulmonary hypertension	10.9	7.3	**< 0.001**	0.13	9.2	8.9	0.291	0.01
Hypothyroidism	22.4	8.4	**< 0.001**	0.40	15.2	15.2	0.940	< 0.01
GORD	31.6	23.4	**< 0.001**	0.18	28.2	28.0	0.593	0.01
Osteoarthritis	30.8	21.8	**< 0.001**	0.21	27.0	26.6	0.368	0.01
Anaemia	15.6	10.9	**< 0.001**	0.14	13.5	13.4	0.730	< 0.01
Falls	9.2	5.3	**< 0.001**	0.15	7.2	7.4	0.602	0.01
Obesity	26.6	23.4	**< 0.001**	0.07	24.5	23.9	0.179	0.01
Prior electrical cardioversion	29.1	34.8	**< 0.001**	0.12	29.2	28.3	0.593	0.01
Medications (%)								
Anticoagulant	84.5	84.0	0.055	0.01	83.4	83.0	0.315	0.01
Beta-blocker	76.6	73.9	**< 0.001**	0.06	75.0	74.6	0.306	0.01
Calcium channel blocker	53.5	48.4	**< 0.001**	0.10	51.1	50.6	0.315	0.01
Flecainide	20.5	14.7	**< 0.001**	0.15	18.7	18.2	0.199	0.01
Amiodarone	25.1	25.6	0.122	0.01	24.6	24.0	0.167	0.01
ACE-inhibitor	25.8	31.8	**< 0.001**	0.13	26.3	25.8	0.187	0.01
Loop diuretic	34.0	28.5	**< 0.001**	0.12	31.1	30.6	0.192	0.01

*ACE,* angiotensin converting enzyme; *GORD,* gastro-oesphageal reflux disease; *PSM,* propensity score matching; *SD,* standard deviation; *SMD,* standardised mean difference

Before PSM, female patients were older (67.2 ± 10.1 vs. 63.8 ± 10.7 years; *p* < 0.001) and more frequently had diastolic heart failure (16.0% vs. 10.2%; *p* < 0.001), hypertension (64.3% vs. 62.9%; *p* < 0.001), hypothyroidism (22.4% vs. 8.4%; *p* < 0.001), pulmonary hypertension (10.9% vs. 7.3%; *p* < 0.001), osteoarthritis (30.8% vs. 21.8%; *p* < 0.001) and anaemia (15.6% vs. 10.9%; *p* < 0.001) (Table 1). Male patients had higher rates of systolic heart failure (16.6% vs. 12.0%; *p* < 0.001), persistent AF (47.0% vs. 41.2%; *p* < 0.001) and chronic ischaemic heart disease (37.0% vs. 29.3%; *p* < 0.001). Female patients were more likely to receive flecainide (20.5% vs. 14.7%; *p* < 0.001) and calcium channel blockers (53.5% vs. 48.4%; *p* < 0.001), whilst male patients were more likely to receive ACE inhibitors (31.8% vs. 25.8%; *p* < 0.001). Prior electrical cardioversion was less frequent in female patients (29.1% vs. 34.8%; *p* < 0.001).

Following PSM, female and male patients were well-matched across all baseline characteristics including cardiovascular comorbidities, with standardised mean differences (SMDs) of < 0.1 for all covariates (Table 1).

Mean follow-up duration after matching was similar between groups (female: 3.1 ± 1.7 years; male: 3.0 ± 1.7 years).

### Primary outcome: AV node ablation

**Fig. 1:**
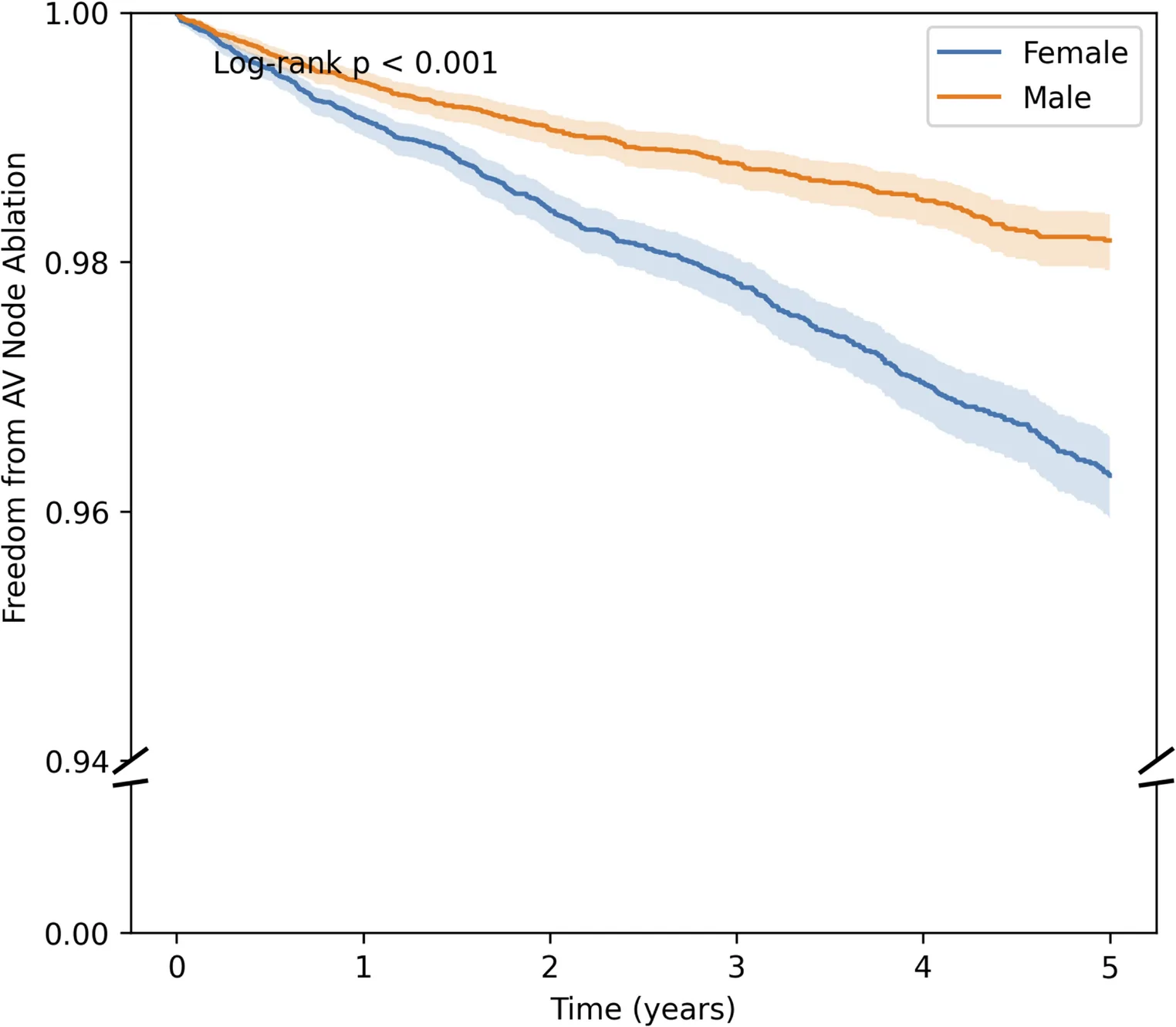
Freedom from atrioventricular node ablation following atrial fibrillation catheter ablation. Kaplan-Meier analysis (after-PSM) for freedom from AVNA following first-time AF catheter ablation, stratified by sex. Female patients demonstrated lower freedom from AVNA over 5-year follow-up compared with male patients. Shaded areas represent 95% confidence intervals

Before PSM, AVNA occurred in 2.5% of female patients and 1.1% of male patients (risk ratio [RR] 2.31, 95% CI 2.07–2.58; *p* < 0.001; Table 2). After matching, this difference persisted, with AVNA occurring in 2.3% of female patients and 1.2% of male patients (RR 1.94, 95% CI 1.68–2.23; *p* < 0.001; Table 3). Kaplan–Meier analysis after PSM demonstrated lower 5-year freedom from AVNA in female patients compared with male patients (96.3% vs. 98.2%; log-rank *p* < 0.001), corresponding to a hazard ratio of 1.90 (95% CI 1.65–2.19; *p* = 0.005; Fig. 1).

**Table 2:** Outcomes before propensity-score matching

	Female	Male	Risk ratio [95% CI]	*p*-value
	(*n* = 28,004)	(*n* = 52,137)		
Primary outcome				
Atrioventricular nodal ablation	2.5%	1.1%	2.31 [2.07–2.58]	**< 0.001**
Secondary outcomes				
Repeat AF ablation	14.7%	14.1%	1.04 [1.01–1.08]	**0.024**
Electrical cardioversion	19.2%	19.3%	0.99 [0.96–1.02]	0.602
Mortality	4.7%	4.7%	0.99 [0.93–1.06]	0.848
Falsification endpoints				
Pneumonia	7.3%	6.1%	1.19 [1.13–1.26]	**< 0.001**
Intestinal obstruction	2.1%	1.7%	1.22 [1.10–1.36]	**< 0.001**

**Table 3:** Outcomes after propensity-score matching

	Female	Male	Risk ratio [95% CI]	*p*-value
	(*n* = 24,377)	(*n* = 24,377)		
Primary outcome				
Atrioventricular nodal ablation	2.3%	1.2%	1.94 [1.68–2.23]	**< 0.001**
Secondary outcomes				
Repeat AF ablation	14.7%	13.3%	1.11 [1.06–1.16]	**< 0.001**
Electrical cardioversion	18.8%	18.2%	1.04 [0.99–1.07]	0.070
Mortality	4.5%	4.9%	0.91 [0.84–0.99]	**0.027**
Falsification endpoints				
Pneumonia	6.8%	6.8%	1.00 [1.94–1.07]	0.885
Intestinal obstruction	1.9%	1.9%	0.99 [0.87–1.12]	0.843

### Secondary outcomes

Secondary outcomes before and after PSM are summarised in Tables 2 and 3, respectively.

Before PSM, repeat AF ablation was slightly more frequent in female patients (14.7% vs. 14.1%; RR 1.04, 95% CI 1.01–1.08; *p* = 0.024), although the absolute difference was small. There were no significant differences in rates of electrical cardioversion (19.2% vs. 19.3%; RR 0.99, 95% CI 0.96–1.02; *p* = 0.602) or all-cause mortality (4.7% vs. 4.7%; RR 0.99, 95% CI 0.93–1.06; *p* = 0.848).

**Fig. 2:**
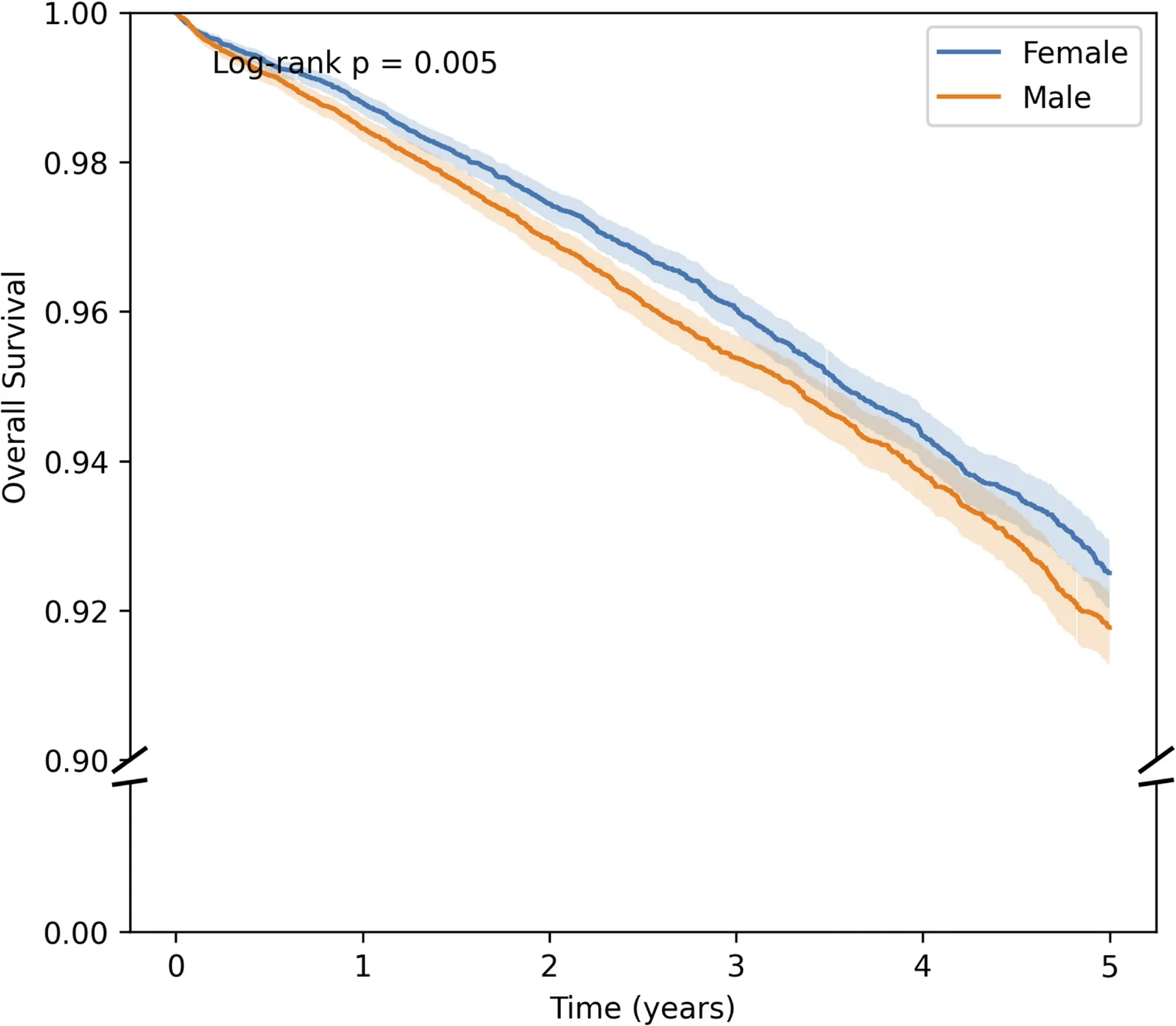
All-cause mortality following atrial fibrillation catheter ablation. Kaplan-Meier analysis (after-PSM) for all-cause mortality following first-time AF catheter ablation, stratified by sex. Female patients had significantly higher overall survival at 5 years compared with male patients. Shaded areas represent 95% confidence intervals

After matching, female patients remained more likely to undergo repeat AF ablation (14.7% vs. 13.3%; RR 1.11, 95% CI 1.06–1.16; *p* < 0.001). Rates of electrical cardioversion were similar between groups (18.8% vs. 18.2%; RR 1.04, 95% CI 0.99–1.07; *p* = 0.070). All-cause mortality was modestly lower in female compared with male patients (4.5% vs. 4.9%; RR 0.91, 95% CI 0.84–0.99; *p* = 0.027), corresponding to a 5-year survival probability of 92.5% and 91.8%, respectively (Fig. 2).

### Falsification endpoints

Before PSM, differences were observed in both falsification endpoints between female and male patients, consistent with baseline imbalance: pneumonia (female 7.3% vs. male 6.1%; RR 1.19, 95% CI 1.13–1.26; *p* < 0.001) and intestinal obstruction (2.1% vs. 1.7%; RR 1.22, 95% CI 1.10–1.36; *p* < 0.001). After matching, no differences were observed in falsification endpoints (pneumonia: 6.8% vs. 6.8%; RR 1.01, *p* = 0.885; intestinal obstruction: 1.9% vs. 1.9%; RR 0.99, *p* = 0.843), supporting the adequacy of matching and reducing the likelihood that the observed differences in AVNA are attributable to residual confounding.

## Discussion

This study is, to our knowledge, the first to describe sex-based differences in escalation to AVNA after first-time catheter ablation for AF (Central Illustration). The primary findings from our analysis are: (i) female patients were more than twice as likely as male patients to undergo AVNA following AF ablation, a difference that persisted after PSM; (ii) female patients were more likely to undergo repeat AF ablation following index ablation; and (iii) all-cause mortality was modestly lower in women, consistent with established sex-based survival differences. Falsification endpoints were balanced after matching, supporting the robustness of these findings.

Overall, these results suggest that, even after adjustment for baseline characteristics, female sex is associated with a higher likelihood of escalation to AVNA following AF catheter ablation, highlighting important sex-based differences in downstream AF management.

### Sex-based differences in rhythm control and escalation to AVNA

Sex differences in the management of AF are well-established. Women are typically older at presentation, with a greater comorbidity burden, higher symptom severity and lower QoL [12]. Despite this, women are less likely to be referred for specialist management and less frequently undergo catheter ablation [2, 13, 14, 16, 17], often resulting in longer AF duration prior to invasive management [14, 15, 40, 41]. This delay may contribute to more advanced atrial remodelling and fibrosis, which are associated with higher rates of AF recurrence following catheter ablation [42–44].

Our findings are consistent with this paradigm. The higher rate of repeat AF ablation observed in women likely reflects greater arrhythmia recurrence in this group, which in turn may lower the threshold for consideration of a definitive rate control strategy, particularly given that women tend to be more symptomatic. The increased use of AVNA in women may therefore represent the cumulative effect of disparities across the AF care pathway, rather than a single decision point.

These observations align with prior studies. In a single-centre retrospective analysis, Calvert et al. reported female sex as an independent predictor of AVNA following repeat AF ablation, with 61.5% of those undergoing AVNA during follow-up being female [41]. Similarly, the ORBIT-AF registry demonstrated higher rates of AVNA among, despite overall low utilisation [7]. Baumgartner et al. reported that, in a large multi-centre cohort of patients undergoing AVNA, approximately 50% were women, and female patients had a more favourable post-procedural course compared with men [45]. Our data complement and extend these findings in a larger, internationally representative population.

### Mechanisms underlying sex-based differences

The higher rate of AVNA observed in women is likely multifactorial, reflecting a combination of anatomical, psychological, procedural and sociological factors.

From a mechanistic perspective, women exhibit distinct patterns of atrial remodelling, including left atrial strain, increased left atrial stiffness and a higher prevalence of low-voltage substrate, consistent with more advanced atrial myopathy [43, 44, 46, 47]. In addition, non-pulmonary vein triggers appear to be more prevalent in women and are associated with poorer outcomes [15, 21, 46, 48, 49]. These factors may limit the effectiveness of pulmonary vein isolation alone and contribute to higher recurrence rates.

Pharmacological considerations may also play a role. Women are more susceptible to adverse effects of antiarrhythmic drugs, including QTc prolongation and bradyarrhythmias, and are more likely to discontinue therapies [11, 17, 18]. This may reduce the available options for rhythm control post-ablation, shifting the balance towards a pace-and-ablate strategy for symptomatic women in whom pharmacological therapy is poorly tolerated.

Beyond these mechanisms, sex-based disparities in symptom perception and social norms are likely to contribute. Women with AF are often more symptomatic and report a lower QoL than men, even when receiving equivalent treatment [7, 8, 50, 51]. It is recognised that female patients have higher rates of cardiac, respiratory, gastrointestinal and neurological symptoms in the first month post ablation [1] and have higher levels of psychological distress, which may amplify symptom reporting and reduce tolerance of arrhythmia recurrence [50, 52]. In addition, women are more likely to undertake the role of primary caregiver for family and children, and as such experience a unique barrier to healthcare access [53]. A combination of increased symptoms and social responsibility may lead female patients to opt for a more definitive strategy when offered.

Finally, clinician-related factors may also contribute. Awareness of sex-based differences in ablation outcomes and recurrence risk may influence clinical decision-making, potentially lowering the threshold for recommending AVNA in women with recurrent AF. In practice, this may manifest as a greater inclination to favour a definitive rate control strategy over repeat ablation in patients perceived to have a lower likelihood of durable rhythm control. In addition, differences in how symptoms are reported and interpreted may influence treatment pathways, with clinicians potentially weighting symptom burden more heavily in women when considering escalation of therapy. While such decisions may be clinically appropriate on an individual basis, they may also contribute to systematic differences in management at a population level. The relative contributions of biological differences, patient preference and clinician decision-making cannot be disentangled in the present analysis.

### Clinical implications

For patients in whom pharmacological rate control is insufficient, or in whom rhythm control strategies are ineffective, not tolerated, or not appropriate, AVNA with permanent pacing is an established and effective strategy, associated with improved symptom control and QoL. In selected patients with heart failure, AVNA combined with CRT reduces hospitalisations and mortality compared with medical therapy alone [28, 31, 33]. These benefits may be particularly relevant in the older, more symptomatic population that characterises many women with AF.

These results have important implications for shared decision-making. Clinicians should recognise that women with recurrent AF after ablation may be more likely to undergo escalation of therapy, and discussions regarding repeat ablation versus definitive rate control should reflect this. Earlier consideration of alternative strategies, including repeat AF ablation, may be appropriate in selected patients, particularly where symptom burden is high, or tolerance of antiarrhythmic therapy is limited.

Importantly, advances in pacing strategies, including conduction system pacing, may mitigate the risk of pacing-induced cardiomyopathy and enhance the safety profile of the pace-and-ablate approach, potentially lowering the threshold for its use in appropriate patients.

More broadly, these findings underscore the need to address sex-based disparities earlier in the AF care pathway. Ensuring timely referral and equitable access to catheter ablation for women may reduce progression to advanced atrial disease and potentially decrease the need for subsequent AVNA. Whether optimisation of ablation strategies can attenuate these observed differences warrants further investigation.

Ongoing studies will further inform the optimal balance between rhythm and rate control strategies in this population. The RHYTHMIC trial (Rate or Rhythm Control in CRT) will directly compare AV node ablation with AF ablation in patients with CRT and sub-optimal biventricular pacing, addressing a key area of clinical uncertainty [54]. The results of this study may help refine patient selection and timing of AVNA, particularly in patients with HF, and provide important context for interpreting the sex-based differences observed in the present analysis.

## Limitations

Our study has several limitations. Firstly, as a retrospective observational analysis, our findings may be subject to confounding, despite the use of PSM. Although falsification endpoints were well-balanced after matching, residual confounding from unmeasured variables – such as symptom severity, patient preference and physician decision-making patterns – cannot be excluded. Therefore, causality cannot be inferred.

Secondly, as with all studies conducted within the TriNetX platform, reliance on ICD-10 and CPT coding for the identification of procedures introduces the potential for misclassification. Although the use of nationally standardised procedural codes for AF ablation and AVNA is unlikely to result in major error, coding inconsistencies across participating institutions may impact the accuracy of individual covariates and outcomes. Procedures performed outside participating TriNetX healthcare organisations may not have been captured, potentially resulting in some under-ascertainment of clinical events. However, any resulting bias would likely be non-differential between female and male patients. The multinational composition of the Global Collaborative Network means that geographical variation in indications for AVNA and repeat AF ablation cannot be adjusted for, and our findings may not be generalisable across all healthcare systems.

Data on initial AF ablation modality (radiofrequency, cryoablation or pulsed field ablation), procedural extent (pulmonary vein isolation alone vs. additional lesions) and post-ablation AAD use and dosage were not available for analysis. These factors may influence arrhythmia recurrence and the decision to escalate to AVNA, and their absence limits interpretation of causal mechanisms. The TriNetX platform does not provide data on patient-reported outcomes such as symptom severity. Moreover, the specific indications driving the choice of AVNA and information about patient preference are not available. Finally, socioeconomic factors that may influence healthcare access and treatment decisions could not be assessed.

## Future considerations

This study is hypothesis-generating and should be regarded as the first step in a more detailed characterisation of sex-based differences in escalation to AVNA after first-time catheter ablation for AF. Prospective studies incorporating patient-reported outcomes, arrhythmia monitoring data and detailed procedural variables are needed to differentiate the mechanisms contributing to the observed sex disparity in AVNA escalation rates. Clinical decision tools to support the timely identification of patients unlikely to benefit from further catheter ablation – and who may be better served by early referral for AVNA – would be meaningful. Whether sex should be formally incorporated into such tools warrants dedicated investigation in prospective studies.

## Conclusion

This study demonstrates that female patients are significantly more likely than male patients to undergo AVNA following first-time AF catheter ablation, with this disparity persisting after robust PSM. Female patients were also more likely to undergo repeat AF ablation, whilst all-cause mortality was modestly lower. These findings highlight important sex-based differences in the downstream management of AF and align with the broader pattern of delayed referral and greater arrhythmia recurrence that characterise women’s experience of AF across the whole management pathway. Further work is needed to understand the mechanisms underlying these differences and to determine whether earlier or more individualised management strategies can reduce disparities in treatment pathways.

## Supplementary Information

Below is the link to the electronic supplementary material.

Supplementary Material 1

## Data Availability

To gain access to the data, a request can be made to TriNetX (https://trinetx.com/), however, a data-sharing agreement is necessary, no patient-identifiable information is obtainable and costs may incur.
