# Supplementary material for: Sex differences in escalation to atrioventricular node ablation following catheter ablation for atrial fibrillation
Source: J Interv Card Electrophysiol. 2026 Jul 1;69(9):2821–30. doi: 10.1007/s10840-026-02395-7 (PMC13769754; doi:10.1007/s10840-026-02395-7)
Supplement: Supplementary file 1 [file 10840_2026_2395_MOESM1_ESM.docx]

**Supplementary Materials – Appendix 1: Diagnostic codes**

*Abbreviations used below:*

- *ICD10CM: International Classification of Diseases, Tenth Revision, Clinical Modification*
- *CPT: Current Procedural Terminology*
- *SNOMED: Systematized Nomenclature of Medicine Clinical Terms*

**Cohort definition**

**Inclusion criteria:**

**Atrial fibrillation catheter ablation:**

- CPT:93656 (Comprehensive electrophysiologic evaluation including transseptal catheterizations, insertion and repositioning of multiple electrode catheters with intracardiac catheter ablation of atrial fibrillation by pulmonary vein isolation, including intracardiac electrophysiologic 3-dimensional mapping, intracardiac echocardiography including imaging supervision and interpretation, induction or attempted induction of an arrhythmia including left or right atrial pacing/recording, right ventricular pacing/recording, and His bundle recording, when performed)

**Exclusion criteria:**

**Atrioventricular node ablation:**

- CPT:93650 (Intracardiac catheter ablation of atrioventricular node function, atrioventricular conduction for creation of complete heart block, with or without temporary pacemaker placement)
- SNOMED:428663009 (Ablation of atrioventricular node)
- SNOMED:175125003 (Percutaneous transluminal ablation of atrioventricular node)

**Permanent pacemaker or cardiac resynchronization therapy:**

- CPT:33206 (Insertion of new or replacement of permanent pacemaker with transvenous electrode(s); atrial)
- CPT:33207 (Insertion of new or replacement of permanent pacemaker with transvenous electrode(s); ventricular)
- CPT:33208 (Insertion of new or replacement of permanent pacemaker with transvenous electrode(s); atrial and ventricular)
- CPT:33224 (Insertion of pacing electrode, cardiac venous system, for left ventricular pacing, with attachment to previously placed pacemaker or implantable defibrillator pulse generator (including revision of pocket, removal, insertion, and/or replacement of existing generator)
- CPT:33225 (Insertion of pacing electrode, cardiac venous system, for left ventricular pacing, at time of insertion of implantable defibrillator or pacemaker pulse generator (eg, for upgrade to dual chamber system) (List separately in addition to code for primary procedure))
- CPT:33249 (Insertion or replacement of permanent implantable defibrillator system, with transvenous lead(s), single or dual chamber)
- ICD10PCS:0JH609Z (Insertion of Cardiac Resynchronization Defibrillator Pulse Generator into Chest Subcutaneous Tissue and Fascia, Open Approach)
- ICD10PCS:0JH639Z (Insertion of Cardiac Resynchronization Defibrillator Pulse Generator into Chest Subcutaneous Tissue and Fascia, Percutaneous Approach)
- SNOMED:429064006 (Implantation of cardiac resynchronization therapy implantable pacemaker)

**Baseline characteristics**

- Persistent atrial fibrillation: ICD10CM:I48.1 (Persistent atrial fibrillation)
- Systolic heart failure: ICD10CM:I50.2 (Systolic (congestive) heart failure)
- Diastolic heart failure: ICD10CM:I50.3 (Diastolic (congestive) heart failure)
- Type 2 diabetes mellitus: ICD10CM:E11 (Type 2 diabetes mellitus)
- Cerebral infarction: ICD10CM:I63 (Cerebral infarction)
- Acute myocardial infarction: ICD10CM:I21 (Acute myocardial infarction)
- Chronic ischemic heart disease: ICD10CM:I25 (Chronic ischemic heart disease)
- Hypertension: ICD10CM:I10 (Essential (primary) hypertension
- Mitral valve regurgitation: ICD10CM:I34.0 (Nonrheumatic mitral (valve) insufficiency)
- Chronic kidney disease: ICD10CM:N18 (Chronic kidney disease)
- Emphysema: ICD10CM:J43 (Emphysema)
- Pulmonary hypertension: ICD10CM:I27.2 (Other secondary pulmonary hypertension)
- Hypothyroidism: ICD10CM:E03.9 (Hypothyroidism, unspecified)
- Gastro-oesophageal reflux disease: ICD10CM:K21 (Gastro-esophageal reflux disease)
- Osteoarthritis: ICD10CM:M15-M19 (Osteoarthritis)
- Anemia: ICD10CM:D64.9 (Anemia, unspecified)
- Falls: ICD10CM:W00-W19 (Slipping, tripping, stumbling and falls)
- Obesity: ICD10CM:E66.9 (Obesity, unspecified)
- Prior electrical cardioversion: CPT:1012978

**Medications:**

- Anticoagulant: Veterans Affairs BL110 (Anticoagulants)
- Beta-blocker: Veterans Affairs CV100 (Beta blockers/related)
- Calcium channel blocker: Veterans Affairs CV200 (Calcium channel blockers)
- Flecainide: RxNorm 4441 (Flecainide)
- Amiodarone: RxNorm 703 (Amiodarone)
- ACE-inhibitor: Veterans Affairs CV800 (Ace inhibitors)
- Loop diuretic: Veterans Affairs CV702 (Loop diuretic)

**Diagnostic codes for outcomes**

**Atrioventricular node ablation:**

- CPT:93650 (Intracardiac catheter ablation of atrioventricular node function, atrioventricular conduction for creation of complete heart block, with or without temporary pacemaker placement)
- SNOMED:428663009 (Ablation of atrioventricular node)
- SNOMED:175125003 (Percutaneous transluminal ablation of atrioventricular node)

**Repeat atrial fibrillation ablation:**

- CPT:93656 (Comprehensive electrophysiologic evaluation including transseptal catheterizations, insertion and repositioning of multiple electrode catheters with intracardiac catheter ablation of atrial fibrillation by pulmonary vein isolation, including intracardiac electrophysiologic 3-dimensional mapping, intracardiac echocardiography including imaging supervision and interpretation, induction or attempted induction of an arrhythmia including left or right atrial pacing/recording, right ventricular pacing/recording, and His bundle recording, when performed)
- CPT:93657 (Additional linear or focal intracardiac catheter ablation of the left or right atrium for treatment of atrial fibrillation remaining after completion of pulmonary vein isolation (List separately in addition to code for primary procedure))

**Electrical cardioversion:**

- CPT:1012978 (Cardioversion, elective, electrical conversion of arrhythmia)
- CPT:92960 (Cardioversion, elective, electrical conversion of arrhythmia; external)
- SNOMED:180325003 (Direct current cardioversion)
- SNOMED:275148007 (External electrode cardioversion)

**Falsification endpoints:**

- Intestinal obstruction: ICD10CM:K56 (Paralytic ileus and intestinal obstruction without hernia)
- Pneumonia: ICD10CM:J18 (Pneumonia, unspecified organism)
